# Book Reviews

**Published:** 1802-05

**Authors:** 


### CRITICAL RETROSPECT

### OF

### MEDICAL AND PHYSICAL LITERATURE.

---

*Nouveaux Elémens de Physiologie*; i. e. New Elements of Phyfiology, by Anselem Richerand. Paris, for Richard, Caille, and Ravier; pp. 700. 8vo. (price 6 livr. 5 f. or about 6s.)

The author of this work has collected into a very fmall compafs the whole knowledge hitherto acquired in phyfiology. Thefe elements are compofed according to the fame view which Haller took, when he made an extract of his great phyficlogy, under the title of *Primæ Lineæ Phyfiologiæ*; and it is intended to give a fhort but accurate account of the ftate of this fcience. We fhall here communicate the plan, which Mr. Richerand has adopted in this work.

Several naturalifts and phyfiologifts have diftinguifhed in men a vegetative or interior, and an animal or exterior life, which divifion the author has alfo adopted; but as it comprifes the functions of the individual only, he has thought proper to modify it, and to eftablifh two claffes of functions: 1. Functions, which tend to the prefervation of the individual. 2. Functions, which tend to the prefervation of the fpecies. The firft clafs is divided into two orders, the firft comprifes the functions by which the food is affimilated with the fubftance of the individual. As the inteftines form a character that fets, as it were, a limit between the animal and vegetable creation, it is natural, that in the enumeration of the genera of this order the author fhould commence with digeftion, the phenomena of which he explains, and that thofe of abforption, circulation, fecretion, and nutrition fhould fucceed. In the fecond order are comprehended the functions, which form the intercourfe of the individual with the objects that furround him. This intercourfe takes place in three ways; by the fenfes, which give notice of the prefence of bodies; by motion, by which we approach, or remove farther from them; by voice and fpeech, which form the communication between man and man, without his having occafion to move from his place. Under the article of the fenfations, he defcribes the organs of fenfe, and explains their manner of action; he gives an account of the brain and nerves; paffing from thence to the human underftanding, he examines the means by which it acquires its knowledge. An enquiry into the ftate of fleeping and waking, dreaming and walking afleep, fympathy and habit, conclude this interefting chapter. In the fecond fub-divifion, which treats of motion, he confiders the organs of motion, that is to fay, the fyftem of bones and mufcles, the parts by which they are united, &c. To this fucceeds an examination of their mode of action, in fitting, ftanding, and progreffion. The third fubdivifion explains the organs of voice, the manner in which it is produced, its different modifications, its defects, &c.

The fecond clafs of functions is alfo divided into two orders. 1ft. That in which the concurrence of both fexes is neceffary, which comprehends conception and generation. 2d. The functions which are confined to women, as geftation or pregnancy, birth, and lactation.

The author has added an Appendix of the phenomena, which appear at the different ages in the two fexes; of temperaments, and of the different races of mankind. This appendix is concluded by an explanation of the decompofitions to which the human body is expofed, when deprived of life, and fubject to the action of air and water, &c.

---

*Memoires sur l'Influence de l'Air et de diverses Substances Gazeuses dans le Germination de diverses Graines*; i. e. Memoirs on the Influence of Air and of the different gazeous Subftances on the Germination of different Seeds; by HUBER and SENEBIER. 1801. Geneve, for Pafchoud. 8vo.

THIS publication has fomething extraordinary in the manner in which it was compofed. Cit. Huber, who is already known by his work on the bees, is blind, but has notwithftanding made the experiments which were fuggefted to him by Cit. Senebier, in order to determine the effects of the different gafses, and particularly of the oxygen gas on germination. The feeds were placed on pieces of wet fponge or flannel, under receivers, which were filled with gas. The following are the principal refults. None of the feeds expofed to azotic gas budded; but did fo, when afterwards placed in the open air. Their germination was accelerated in pure oxygen gas; but the plants were more vigorous, when it contained a fmall quantity of carbonic acid. In this experiment the carbon of the feed combining itfelf with the oxygen, forms acid carbonic gas. The feeds germinated in artificial atmofpheric air as well as in common atmofpheric air. The moft favourable proportion for germination is three meafures of azote or hydrogen for one of oxygen. Seeds placed in azotic gas did not germinate, even when a tolerable quantity of oxygen was added by degrees, but they germinated very well when the oxygen was added all at once. The caufe of this difference is, that in the firft cafe the oxygen is continually employed in receiving from the feed the difengaged carbon, fo that there does not remain enough to vivify it; whereas, when it is all at once admitted, there is fufficient for both purpofes.

The feeds did not germinate at all in carbonic acid gas, nor in pure hydrogen: One lettuce feed abforbs in germination a quantity of oxygen, equal almoft to .26 milligrammes of water; but it does not germinate till the oxygen forms at leaft one-eighth of the atmofphere in which it lies. A furplus of carbonic acid is more hurtful to germination than that of azote, and a fuperabundance of of azotic gas more noxious than that of hydrogen. When feeds germinate in hydrogen gas, the carbon of the feed diffolves in it, and combines itfelf very intimately with it.

The vapours of fulphuric ether in a receiver full of atmofpheric air hinder the germination of feeds, without altering the quantity of oxygen. The vapours of camphor, oil of turpentine, affafœtida, and fal ammoniæ, had the fame effect. Putrifying bodies prevent germination by the quantity of carbonic acid they emit. It appears from the above facts, that oxygen is indifpenfable for germination, that it ferves to take away from the feed the carbonic acid which is difengaged by fermentation; this rule, however, feems not to be without exception. In fact, peas germinated in water, which had been deprived of air by all poffible means, at whatever depth they were immerfed.

Beans, lentils, the feeds of fpinage, lettuce, and corn germinated under water with more or lefs facility, but better in water that contained oxygen, than in that deprived of it. They however, did not germinate in water containing carbonic acid. Acids retard more or lefs germination. The air emitted from peas under pure water, is a mixture of carbonic acid and carbonated hydrogen.

Peas germinated in pure hydrogen gas, in airs in which other feeds had already germinated, and they exhaufted the reft of the oxygen that was left in them. They have alfo germinated in azotic gas, but they did not germinate in oil, though, if put into oil after having fwelled in water, they germinated very well.

Thefe facts are much in favour of the decompofition of water in germination, and confequently in vegetation in general.

---

*Traité de Mineralogie*; i. e. Treatife of Mineralogy, by Citizen HAUY, publifhed by the Council of the Mines 5 vol. each of which contains 86 plates. Paris 1801, for Louis.

THIS excellent work, of which the Mineralogifts have long been in expectation, anfwers fully the requifites of a complete Treatife of Mineralogy, digefted with method, clearnefs, care, and exactnefs, by a man, whofe numerous obfervations have already fo much contributed to the advancement of that fcience, and who ranks fo very high amongft the Mineralogifts. It is only neceffary to read a few pages, to difcover the great difference between this work and the hafty compilations with which that fcience has of late overflowed. In the firft volume, and part of the fecond, Citizen Haüy explains the properties common to minerals in general; he develops the theory of many properties, which ought to ferve as characters to diftinguifh minerals, characters fo much the better chofen as they are effential to the nature of thefe fubftances. In the fame volume, we find the explanation of his theory of cryftallization and the enumeration of the phyfical, chemical, and geometrical characters that are obvious in minerals. Cit. Haüy has prefented, in tables, the claffes of minerals, eftablifhed after the different characters, each confidered exclufively; he has feparated from the theory of cryftallization itfelf, the geometrical part. In this Introduction he makes likewife likewife known the method he has adopted, and the reafons which induced him to prefer this method, founded only upon what is well known, to a fyftem more complete, but which is frequently under the neceffity of propofing as certain what is really nothing but conjecture. He has not therefore hefitated to place in the Appendix, all thofe fubftances which are not fufficiently pure to be regarded as fpecies, or fufficiently known to be placed methodically. In the fame preliminary Difcourfe, he gives the rules he has followed in eftablifhing his Nomenclature, which is as fimple, methodical, and uniform, as the ftate of the fcience would allow.

The fpecies are defcribed in the following volumes, and the hiftory of each given as exactly as poffible; they confift chiefly in the fynonima, the characters, its phyfical, chemical, and geometrical properties; their geographical and geological fituation, and their ufes. The greateft part of the Notes refpecting the geological fituation, was by Cit. Dolomieu; thofe relating to the medicinal and chemical ufes are from Halle, Chaptal, and Vauquelin. The figures annexed to this work, reprefenting the various forms of each fpecies, are made with a care and exactnefs never before attained in this line of drawing; they are done with much tafte by the Members of the Infpection of Mines, after the ftricteft rules of projection.

---

*Anatomie Générale appliquée à la Physiologie et à la Medicine*; i. e. General Anatomy applied to Phyfiology and Medicine, by Xav. Bichat, &c. 4 vol. in 8vo. Paris year 10, (1801) for Broffon, Gabon, and Co. Price 16 livres, 50 centimes.

The work here announced, is, according to the author, entirely new in three refpects; that of the plan, of the doctrine, and of the facts it contains. Cit. Bichat admits twenty-one fpecies of fimple texture, (tiffu), each of which enjoys the fame properties, has the fame ftructure whatever may be their figure or combination with the organs of which they form a part. It is a fort of analyfis in which the textures or telas, regarded as the elements of the human body muft be fucceffively ftudied under the name of fyftems, an idea which the author propofes to make known in another work, of which the firft volume has already appeared, under the title of *Descriptive Anatomy*. The organic fyftems form two claffes. In the firft are arranged the textures (tiffus), which form the bafes of all our parts, and which affift mutually in the formation of all the apparatus of life, as, the tela cellulofa, the arterial, veinous, the exhaling, abforbing, and nervous membranes. The fecond clafs comprifes the telas, whofe exiftence is as it were infulated, which have a fixed place affigned, as the fyftems of the bones, cartilages, the medullary, fibrous, mufcular, glandulous, mucous, ferous fyftems, &c. The doctrine of this work confifts effentially, *first*, in the analyfis of the properties and phenomena which the human body prefents while alive, confidered in the ftates of health, difeafe, and in the means the phyfician employs to bring back the functions to their their primitive ſtate. *Secondly*, in the diſtinction of the two ſorts of life, animal and organic.

There are a great number of new facts which the author confeſſes to have obſerved in his experiments on living animals, and a great part of them are drawn from his reſearches on the tiſſue by means of air, caloric, water, &c. and principally from his ſtudy of the fineſt parts of anatomy.

